# Comparing MCMC and INLA for disease mapping with Bayesian hierarchical models

**DOI:** 10.1186/2049-3258-73-S1-O2

**Published:** 2015-09-17

**Authors:** Tom De Smedt, Koen Simons, An Van Nieuwenhuyse, Geert Molenberghs

**Affiliations:** 1 Wetenschappelijk Instituut Volksgezondheid / KU Leuven (Leuvens Biostatistiek en Statistische Bioinformatica Centrum), Brussels, Belgium; 2 Wetenschappelijk Instituut Volksgezondheid, Brussels, Belgium; 3 K.U. Leuven, Leuven, Belgium

## Introduction

Bayesian hierarchical models with random effects are one of the most widely used methods in modern disease mapping, as a superior alternative to standardized ratios. These models are traditionally fitted through Markov Chain Monte Carlo sampling (MCMC). Due to the nature of the hierarchical models and random effects, the convergence of MCMC is very slow and unpredictable. Recently, Integrated Nested Laplace Approximation was developed as an alternative method to fit Bayesian hierarchical models of the latent Gaussian class.

## Materials and methods

In order to compare MCMC and INLA for disease mapping in terms of accuracy and computational burden, we selected the Bayesian hierarchical model with conditional autoregressive priors. Belgian cancer mortality data on breast cancer and acute childhood leukaemia from 2003 until 2010 and a simulation study are used to compare both methods. Disease counts were simulated for each Belgian municipality based on its respective population, and three simulation parameters: the base risk of the disease and a non-spatial and a spatial component of the relative risk. Each simulation parameter has three different settings, leading to 27 different simulation scenarios, and each scenario was simulated 10.000 times.

## Results

The simulation study clearly shows that INLA is equivalent to MCMC for parameter estimation in disease mapping studies. Only when the disease is statistically very rare does INLA provide worse estimates and credible intervals. We also see that when the spatial component is higher, the estimates and credible intervals of both methods become worse. The overall computational burden of INLA was 42 times lower. MCMC and INLA performed similarly for actual cancer mortality data.

## Discussion

Both the simulation study and the breast cancer case study show that INLA is a suitable alternative to MCMC for disease mapping. Great care should be given when the disease is statistically rare.

